# P08-01 Demographic, social, and environmental factors predicting Danish children's greenspace use

**DOI:** 10.1093/eurpub/ckac095.114

**Published:** 2022-08-29

**Authors:** Jan Arvidsen, Tanja Betinna Schmidt, Søren Præstholm, Søren Andkjær, Anton Stahl Olafsson, Jonas Vestergaard Nielsen, Jasper Schipperijn

**Affiliations:** 1 Department of Sports Science and Biomechanics, University of Southern Denmark, Odense M, Denmark; 2 Department of Geosciences and Natural Resource Management, University of Copenhagen, Copenhagen, Denmark

**Keywords:** children, greenspace use, accessibility of greenspace, parental barriers, modifiable factors

## Abstract

**Background:**

Evidence suggest that greenspace use can be associated with children's physical, mental, social health, and well-being (Tillmann et al., 2018; Mygind et al., 2019; Mygind et al., 2021). Greenspace can facilitate a wide range of low-cost activities and the availability of greenspace is frequently linked to increased levels of recreational physical activity. Accordingly, contemporary children's declining greenspace use prompts a need to understand the factors that affect frequency of use.

**Methods:**

Aiming to determine to what extent demographic, environmental and social factors predict greenspace use for school-aged (6-15-year-old) children in Denmark, a national online survey was distributed to parents of 10.000 0-15-year-old children. From a total of 4772 responses a sub-sample of 3171 responses from parents of school-aged children was included in the analysis for this study. The aim was addressed by answering the following research questions: 1) How often do Danish children use greenspaces? 2) What demographic differences in greenspace use are present? 3) How do social and environmental factors predict greenspace use?

**Results:**

Responses from the 3171 parents showed that 49.5% of the children used greenspace almost every day during the summer season. Multivariate binary logistic regression analysis showed that the number of types of greenspaces within walking or cycling distance from home was a strong predictor for daily use. Social factors (parental concern and encouragement) also predicted use, but less so. Geography and child age were the only demographic predictors for using greenspace almost every day.

**Conclusions:**

Findings from the present study suggest that providing opportunity for choosing between various types of greenspaces within walking or cycling distance might be an effective way to stimulate children's use of greenspace. Also, increased parental awareness of their role in children's outdoor spatial behaviours might further greenspace use among children.

